# Preconception Care and Genetic Screening: A Global Review and Strategic Perspectives for Implementation in Bulgaria

**DOI:** 10.3390/children12111538

**Published:** 2025-11-14

**Authors:** Eleonora Hristova-Atanasova, Martina Micallef, Julia Stivala, Georgi Iskrov, Elitsa Gyokova

**Affiliations:** 1 Department of Social Medicine and Public Health, Faculty of Public Health, Medical University of Plovdiv, 4002 Plovdiv, Bulgaria; georgi.iskrov@mu-plovdiv.bg; 2 Faculty of Medicine and Surgery, University of Malta, 2080 Msida, Malta; martina.micallef.23@um.edu.mt (M.M.); julia.stivala.23@um.edu.mt (J.S.); 3 Department of Obstetrics and Gynecology, Faculty of Medicine, Medical University—Pleven, 5800 Pleven, Bulgaria; elitca.gaokova@mu-pleven.bg; 4 Obstetrics Clinic, UMHAT “Saint Marina” Pleven, 5800 Pleven, Bulgaria

**Keywords:** preconception care, carrier screening, reproductive health policy, child health, Bulgaria, preventive medicine

## Abstract

**Highlights:**

**What are the main findings?**

• A structured narrative synthesis maps international models of preconception care (PCC) and preconception genetic screening to the Bulgarian context, identifying system levers (NHIF/primary care), gaps in reimbursement and genetics capacity, and feasible policy steps.
• Acceptance of PCC and genetic screening is shaped by cultural, religious, and community norms (with Israel as an instructive comparator); rights-based safeguards—voluntariness, informed consent, confidentiality, and non-discrimination—are essential.
• Cross-cutting domains—mental health, environmental/occupational exposures, and men’s preconception health—should be integrated to improve uptake and equity.

**What is the implication of the main finding?**

• Policymakers can embed PCC into primary care with clear guidelines, provider training, and NHIF-backed financing, using a phased, voluntary approach to expanded carrier screening supported by culturally competent counselling.
• Equity-focused outreach (including underserved/rural communities and partner/men’s involvement), together with digital self-assessment tools and routine audit/registry, can scale implementation while safeguarding human rights.

**Abstract:**

**Background:** Preconception care (PCC) is a key element of preventive reproductive health, aiming to optimise maternal and child outcomes by addressing biomedical, behavioural, psychosocial, and genetic risks before conception. International frameworks provide clear guidance, yet implementation in many low- and middle-income countries remains inconsistent. **Methods:** A structured narrative review was conducted across PubMed, Web of Science, Cochrane Library, and Google Scholar, focusing on literature published between 2010 and 2025. Eligible sources included empirical studies, clinical guidelines, policy documents, and high-quality grey literature from health authorities. Quality, relevance, and applicability were assessed, with particular emphasis on European and Bulgarian contexts. **Results:** Evidence from diverse settings demonstrates that PCC interventions—such as chronic disease management, vaccination, lifestyle optimisation, and expanded carrier screening (ECS)—can reduce adverse pregnancy outcomes and prevent severe genetic disorders. Effective international models integrate PCC into primary care, leverage digital health tools, and ensure equitable access through public funding. In Bulgaria, PCC remains underdeveloped: genetic screening is not part of routine care, there are no national guidelines or surveillance systems, and only ~4% of women initiate folic acid supplementation before pregnancy. NGOs and EU-funded digital initiatives provide partial outreach but cannot replace state-supported services. **Conclusions:** Bulgaria urgently requires a coordinated national PCC strategy, incorporating standardised guidelines, provider training, digital platforms, and phased ECS introduction. Strengthening PCC delivery can reduce preventable maternal and neonatal morbidity, advance reproductive justice, and enhance the long-term sustainability of public health systems. These findings support the development of a publicly funded, guideline-driven national PCC strategy with phased introduction of expanded carrier screening under NHIF to improve equity and long-term system sustainability.

## 1. Introduction

The main goal of preconception care (PCC) is to enhance maternal, neonatal, and long-term child outcomes while minimising preventable pregnancy-related complications [1,2,3]. In other words, PCC is a collection of preventive health interventions that are administered prior to conception or between pregnancies. It is designed to address modifiable biomedical, behavioural, genetic, and psychosocial risk factors that impact reproductive outcomes, thereby guaranteeing a healthier start for the next generation [4,5].

PCC incorporates interventions such as structured health promotion, vaccination, nutritional optimisation, chronic disease management, and STI prevention, which can be delivered universally or customised to individual risk profiles [3,6].

PCC is acknowledged as a critical element of the prevention of intergenerational diseases and reproductive health by international organisations like the World Health Organisation (WHO). The WHO PCC action plan emphasises the importance of ensuring that marginalised populations have access to services in a fair manner [7,8]. In low-resource contexts, however, social determinants—such as financial constraints, rural inaccessibility, and low health literacy—continue to impede uptake [9,10,11]. Systemic challenges also confront healthcare professionals, including inadequate training, incomplete incorporation of PCC into primary care pathways, and the absence of standardised guidelines [12,13]. Consequently, the provision of PCC remains fragmented, despite the substantial evidence of its cost-effectiveness and its potential to prevent adverse infant health outcomes.

The implementation of PCC in Bulgaria is limited and poorly integrated. Most women of reproductive age obtain information from informal or non-medical sources, and awareness among women of reproductive age is minimal. General practitioners (GPs) are perceived as reactive rather than proactive [14]. The absence of reliable, readily accessible resources is reflected in the interest in a centralised digital platform, and structured counselling is very rare.

At present, Bulgaria does not have a unified national PCC strategy, and preconception counselling is not systematically integrated into reproductive health services. This disparity is indicative of the absence of formal guidelines, the absence of public funding mechanisms, and the minimal integration with primary care, which result in the underutilisation of most preventive opportunities and the exacerbation of health inequalities.

This structured narrative review critically evaluates international implementation models, synthesises global evidence on PCC and genetic screening, and identifies gaps and policy implications for the expansion of PCC within the Bulgarian healthcare system.

## 2. Materials and Methods

This narrative review adhered to the structured review framework established by Templier and Paré [15] and sought to deliver a thorough and critical synthesis of international practices in PCC and genetic screening, highlighting their significance within the Bulgarian healthcare system. A comprehensive search was performed in Web of Science, MEDLINE/PubMed, Google Scholar, and the Cochrane Library utilising combinations of specified keywords including preconception care, genetic counselling, carrier screening, and premarital screening, incorporating Boolean operators and MeSH terms where applicable. The search encompassed papers from January 2010 to May 2025 to obtain the most current facts and policy advancements.

Studies were included if they pertained to PCC, reproductive health policy, genetic counselling, or carrier screening, and if they offered empirical, conceptual, or policy-relevant information. Eligible sources included peer-reviewed publications, clinical guidelines, government reports, and high-quality grey literature pertaining to human populations; animal research, case reports, conference abstracts, and non-English sources were omitted. The choice to restrict the inclusion to English-language publications was a pragmatic limitation; however, it is recognised as a constraint, especially with national-level data from Bulgaria and other non-English-speaking nations.

All pertinent study designs were evaluated, encompassing cross-sectional, cohort, case–control, prospective observational, qualitative research, and systematic or scoping reviews, in addition to rigorous economic evaluations and model-based analyses. Each source underwent a careful evaluation for methodological rigour, relevance, and applicability in various healthcare contexts. Possible sources of bias encompassed publication bias, stemming from the increased probability of disseminating good findings, linguistic bias, and the inconsistent quality of grey literature. Consequently, government and Non-Governmental Organization (NGO) reports were assessed for transparency, sample representativeness, and methodological rigour prior to inclusion.

Data regarding PCC policies, intervention kinds, coverage rates, and health outcomes were extracted through a structured methodology and synthesised thematically. International comparisons utilised the most recent data available; the label “No data” in summary tables indicates a specific absence of publicly accessible or English-language material and should not be construed as evidence of the nonexistence of policy or action. All evidence was contextualised for Bulgaria by considering the country’s healthcare system structure, financing mechanisms, demographic trends, and current maternal-child health programs. In instances when national-level quantitative data was insufficient, regional or qualitative research was incorporated, explicitly designated as non-representative.

## 3. Global Standards and Best Practices in PCC

PCC has become fundamental to maternal and newborn health, seeking to mitigate preventable difficulties by treating biological, behavioural, psychological, and genetic concerns before conception [6]. The WHO officially acknowledged PCC, resulting in the 2013 PCC Policy Framework that advocates for a comprehensive approach integrating chronic disease management, nutritional enhancement, vaccination, lifestyle alteration, mental health, and environmental risk reduction [7,8]. The Lancet Series on PCC further underscored its incorporation into universal health coverage and primary care [16].

Despite the substantial evidence, worldwide implementation is inconsistent. Affluent nations with robust primary care frameworks like the Netherlands and Sweden effectively integrate structured patient-centred treatment with computerised instruments for risk assessment, enhancing early diagnosis and patient involvement. In contrast, low- and middle-income countries (LMICs) encounter restricted provider training, absence of national guidelines, and ongoing socioeconomic and geographic inequalities [9,10].

A fundamental component of PCC is the early identification of risks. Structured evaluations enable clinicians to avert high-risk pregnancies and enhance newborn outcomes. An Indian study indicated that almost one-third of births were classified as high risk due to chronic illnesses like hypertension and hypothyroidism, with a significant correlation to maternal age and socioeconomic position [17]. International standards, including those from American College of Obstetricians and Gynaecologists (ACOG) [18], recommend providing PCC evaluations to all individuals of reproductive age, irrespective of their fertility objectives. The increasing utilisation of telehealth, mobile applications, and AI-enhanced reproductive life planning offers potential for expanded access, although digital disparities persist as a challenge [19].

Optimisation of chronic diseases and preventive health treatments is essential. Effective management of diabetes and hypertension prior to conception can significantly decrease the incidence of preterm birth, stillbirth, and foetal growth restriction [20,21]. Early optimization of chronic conditions such as diabetes, hypertension, and autoimmune disorders before conception substantially reduces the risk of preterm birth, intrauterine growth restriction, and gestational complications [16,22,23]. Prompt immunisation and folic acid supplementation continue to be economically viable methods for preventing neural tube abnormalities and early newborn morbidity [24,25]. Behavioural interventions, including smoking cessation and healthy dietary habits, are essential components of PCC [26,27]. Digital and community-based educational programs can enhance women’s engagement and awareness regarding vaccinations such as rubella and varicella [28,29]. Evidence also supports the role of multivitamin supplementation in improving fertility and reproductive outcomes [30]. These approaches directly enhance infant health outcomes by mitigating problems that lead to newborn mortality.

Implementation pathways that integrate mental health, environmental/occupational risk assessment, and men’s health—supported by coordinated, adequately financed service models—are essential for equitable access to preconception care and genetic screening [10,31,32,33].

The prevention and management of sexually transmitted infections is another essential element. Infections like chlamydia and gonorrhoea are associated with infertility, ectopic pregnancy, and negative perinatal outcomes. The WHO advocates for routine screening and dual-protection counselling, while implementation significantly differs, especially in resource-constrained areas [34,35,36,37].

The adoption of PCC across the European Union remains highly variable and fragmented. Northern and Western European countries generally demonstrate broader coverage, often embedded in national maternal or reproductive health policies, with some integration of digital tools and risk assessment platforms. In contrast, several Central, Eastern, and Balkan states lack comprehensive national PCC protocols, resulting in inconsistent service delivery and inequitable access (Table 1) [14,38,39,40,41,42,43,44,45,46,47,48,49,50,51,52,53,54,55,56,57,58,59,60,61,62,63,64,65,66,67,68,69,70,71,72,73,74,75,76,77,78,79,80,81,82,83,84,85,86,87,88,89,90,91,92,93,94,95,96,97,98,99,100,101,102,103,104,105,106,107,108,109,110,111,112,113,114,115,116,117,118,119,120,121,122,123,124,125,126,127,128,129,130,131,132,133,134,135,136,137,138,139,140,141,142,143,144,145,146,147,148,149,150,151,152,153,154].

International delivery models show notable diversity. In the Netherlands, PCC is mainly delivered by independent primary-care midwives supported by national digital self-assessment tools; protocol use is inconsistent and uptake remains low, prompting calls for stronger local collaboration. Finland relies on public-health nurses in municipal centres within a universal, family-centred model (including partners), while Denmark mirrors a midwife-led primary-care approach with GP/OBGYNs collaboration; both employ digital tools and campaigns [44,64,65,66,67,68,69,76,77,78,79,80,81,82,125,126,127,128,129,130,131]. In Belgium, regional PCC programmes actively promote folic acid supplementation and lifestyle counselling [43,44,45,46,47,48]. In Nordic countries such as Finland and Denmark, PCC is universally available through established maternity and child health clinic systems, with midwives playing a central role [64,65,66,67,68,69,76,77,78,79,80,81,82]. By contrast, in parts of Southern and Eastern Europe, PCC remains integrated only partially within maternal health services, with delivery often reliant on local initiatives, NGOs, or targeted outreach to vulnerable populations [14,49,50,51,52,53,54,55,56,57,58,59,93,94,95,96,97,98,120,121,122,123,124,134,135,136,137,138,139,140,141,142,143,144,145,146,147,148,149,150].

Outside the EU, approaches also differ considerably. In the UK, national NICE guidelines cover a wide range of PCC risk factors, yet implementation in primary care remains suboptimal due to time constraints and low public awareness [36,37]. In the United States, service provision is affected by disparities in insurance coverage [36]. In parts of the Middle East, compulsory premarital screening programmes have contributed to a reduction in certain genetic disorders, though these approaches raise ethical concerns, including potential limitations on personal autonomy and risks of stigma [9,36]. In many low- and middle-income countries, community outreach and mobile health clinics remain essential for reaching underserved groups, though sustainability depends on stable funding and strong policy support [9,37].

## 4. Genetic Counselling and Carrier Screening in PCC

Genetic counselling and carrier screening are critical elements of PCC, designed to avert serious inherited childhood disorders, such as autosomal recessive and X-linked conditions. Their significance is growing because of increasing maternal age, the broader application of assisted reproductive technologies, and ongoing consanguineous marriages. Genetic disorders are a leading cause of infant mortality and paediatric morbidity, and estimates suggest that up to 6% of global live births may be affected, although this proportion varies depending on definitions, diagnostic methods, and population characteristics [156,157].

Ethnicity-based carrier screening has traditionally focused on populations with established risks, including Ashkenazi Jews and Mediterranean communities. This approach, although cost-efficient, does not effectively identify numerous at-risk couples within diverse or mixed populations [158,159]. Expanded carrier screening (ECS), facilitated by next-generation sequencing, permits the concurrent examination of numerous genes regardless of family history or ancestry, thereby enhancing diagnostic equity. Large-scale genomic studies estimate a reproductive risk of approximately 1 in 337 conceptions for severe childhood-onset disorders, though this figure can vary depending on the screening panel and population studied [158]. International experience demonstrates that pilot ECS programs in Asia and the Middle East have successfully identified at-risk couples and informed reproductive choices [159,160]. However, willingness-to-pay studies reveal that cost and ethical considerations remain significant barriers, particularly in low-resource settings [161,162]. Premarital screening programs can reduce the burden of recessive disorders but raise questions related to human rights and social stigma [163,164].

ECS encounters considerable obstacles to implementation, despite its advantages. High testing costs, lack of standardisation among commercial panels, complexity in interpreting variants of uncertain significance, and absence of public reimbursement hinder its incorporation into routine care. ECS is predominantly offered in private fertility clinics, which raises concerns regarding equity and reproductive justice.

Ethical considerations play a crucial role in preconception genetic screening. Informed consent should clearly convey the voluntary nature, scope, and limitations of testing, as well as potential psychological effects, including depression, shame, and stress related to decision-making. Confidentiality and its implications for biologically related family members should be considered in relation to the principles of autonomy and beneficence [165,166].

From a public health perspective, modelling studies suggest that ECS can be cost-effective, as preventing even a single case of certain severe conditions, such as cystic fibrosis or thalassaemia, may offset the costs of multiple screening programmes; however, this potential depends on local disease prevalence, healthcare costs, and implementation models [156]. A phased implementation, beginning with high-risk groups and partial reimbursement, alongside health literacy programs, is frequently proposed to achieve a balance among equity, ethics, and financial sustainability.

The incorporation of PCC into standard health systems is obstructed by various systemic, provider-level, and population-level challenges, in addition to genetic screening. The lack of national PCC policies standardised clinical guidelines, and sustainable financing at the system level restricts widespread adoption [1]. Barriers to primary care encompass inadequate provider training, ambiguous role definitions, and restricted consultation time, further exacerbated by low patient demand and awareness [167,168]. Population-level uptake is limited by low health literacy, geographic disparities, and sociocultural factors, such as gender norms that assign reproductive responsibility mainly to women and religious beliefs that may hinder acceptance of genetic screening [20,161].

## 5. Bulgarian Context

In Bulgaria, PCC is inadequately developed and insufficiently integrated into the public healthcare system. GPs hold formal responsibility for preconception counselling; however, its implementation is constrained by the lack of national clinical guidelines, inadequate provider training, and the absence of structured pathways and financing mechanisms [14]. The National Programme for Improving Motherhood and Child Health, focuses predominantly on pregnancy outcomes, neglecting preconception risk mitigation. Additionally, family planning consultations are among the lowest in the European Union, despite adverse demographic trends [169,170].

The awareness and uptake of PCC at the population level are minimal. Qualitative research suggests that numerous Bulgarian women associate the onset of pregnancy with a positive test result, while persisting in risk behaviours such as smoking, alcohol consumption, and irregular nutrition throughout early gestation [157]. Despite relatively high awareness of folic acid supplementation, only a small proportion of women report using it prior to conception; available Bulgarian estimates, such as ~4% in one study, are based on limited samples and are not nationally representative [157,171]. Globally, periconception folic acid supplementation is recognised as a highly cost-effective measure for preventing neural tube defects and improving early pregnancy outcomes [26]. In Bulgaria, this low uptake, together with observed behavioural patterns during early pregnancy [172], points to a lack of structured counselling and the absence of consistent national health campaigns. Rural and socioeconomically marginalised populations face additional barriers, including low health literacy and restricted access to primary care, while men are rarely engaged in preconception initiatives, reflecting persistent gendered norms in reproductive health [159,172].

In Bulgaria, there is no national protocol or public funding for preconception carrier screening; available genetic services are mainly provided in academic centres or private clinics, limiting access for much of the population [173]. The absence of these services persists despite international guidance that includes genetic risk assessment as part of comprehensive PCC, with International Federation of Gynaecology and Obstetrics (FIGO) recommending carrier screening within its PCC checklist [25] and ACMG advising that screening for autosomal recessive and X-linked conditions be offered to all individuals planning a pregnancy or who are pregnant [160]. WHO’s PCC framework also highlights targeted genetic screening in high-prevalence settings as part of a broader preventive strategy [8,9].

Non-governmental organisations play a significant complementary role in addressing systemic deficiencies, delivering mobile sexual and reproductive health services, contraception, and STI testing, particularly in underserved areas [174]. The Bulgarian Family Planning and Sexual Health Association (BFPA) has led impactful initiatives in Roma communities and rural regions, including cross-border advocacy and youth-focused campaigns such as “Promoting Sexual and Reproductive Health Services and Human Rights for Youth” [175]. BFPA has also contributed to the institutionalisation of health mediation in Roma communities, training peer educators to improve access to primary healthcare and family planning [176].

Current BFPA initiatives (“Learning Action Partnership”, “Play & Learn”, and “Launching Young Roma Advocates”) demonstrate the organisation’s sustained role in enhancing reproductive health literacy, promoting contraceptive use, and facilitating preconception counselling among marginalised groups [177,178,179,180].

The lack of systematic monitoring and evaluation represents a critical barrier. Bulgaria does not maintain a national registry for PCC delivery, folic acid supplementation, or risk assessment prior to conception. Most available studies are small scale, localised, and primarily urban, which limits their national representativeness [14,157].

## 6. Discussion

Bulgaria, Romania, and Greece are among the few countries in the European Union lacking a formal PCC strategy, while Hungary and Poland have commenced pilot programs for folic acid supplementation and targeted carrier screening. Comparative evidence suggests that effective approaches in Eastern Europe utilise low-cost educational campaigns, mobile outreach teams, and digital platforms, indicating potential strategies for adaptation within the Bulgarian context with limited financial resources. Our added analysis explicitly maps international models to Bulgarian feasibility and ethics, offering a pragmatic policy pathway rather than a generic review.

This review underscores the ongoing disparity between international PCC frameworks and their execution in LMICs contexts. Global evidence indicates that PCC interventions, including lifestyle modification, chronic disease management, and genetic screening, effectively reduce adverse maternal and neonatal outcomes [6,159]. Despite a clear consensus on the conceptual model, operationalisation varies significantly across healthcare systems.

Bridging the global evidence to Bulgaria requires accounting for NHIF financing, primary-care gatekeeping, and uneven genetics capacity. We therefore prioritise low-cost, digitally supported pathways and phased ECS under public funding. PCC has not been formally incorporated into national health policy or primary care in Bulgaria. In contrast to the Netherlands and the UK, where PCC is integrated into routine services with digital support, Bulgaria is deficient in standardised clinical pathways, financial incentives, and coordinated coverage [181,182]. These cross-country differences underscore the need for context-specific adaptation for Bulgaria, while learning from comprehensive PCC infrastructures where they exist (e.g., universal or digitally supported primary-care models). Inadequate provider training, fragmented service delivery, limited public awareness, and dependence on intermittent NGO initiatives are among the systemic barriers that persist. It is particularly concerning that preconception genetic screening is not implemented, as international guidelines recommend its implementation in high-risk groups, such as those with a higher incidence of hereditary illnesses or increased consanguinity [7,8,166]. Despite the presence of limited services in academic centres, accessibility remains a significant issue for most women, leading to preventable morbidity and compromising reproductive autonomy. Access to PCC in LMICs is often constrained by low awareness, cultural barriers, and insufficient provider training [183,184,185]. Health system weaknesses, including poor integration of pre-pregnancy services and limited utilization of pre-marital clinics, further impede uptake [186]. Primary healthcare practitioners in LMICs frequently report gaps in knowledge of genetics and preconception interventions [187,188,189,190]. Digital health tools and targeted public communication strategies constitute an underutilised opportunity. Teleconsultation, mobile applications, and AI-driven risk assessment tools have shown potential to enhance engagement in underserved areas [19,160]. In Bulgaria, numerous women depend on non-medical online resources; such solutions may address systemic deficiencies, contingent upon equitable digital access and clinician involvement. We contrast leading models on coverage, workforce, digital supports, and ethical safeguards to indicate transferability to Bulgaria. Table 2 summarises strengths, limitations, and likely implementation requirements under Bulgarian constraints.

The gendered framing of PCC necessitates focused examination. Reproductive health is frequently perceived as solely a woman’s responsibility; however, paternal factors, including age, obesity, and exposure to toxins, significantly affect fertility and offspring outcomes [20,23]. Effective PCC strategies should actively involve men in public messaging and service delivery. Comparative policy modelling in accordance with WHO and Horizon Europe frameworks can enhance the creation of scalable and culturally suitable PCC models for Bulgaria.

## 7. Cultural, Religious, and Human Rights Considerations

Comparative evidence from Israel, spanning Jewish and Israeli Arab groups as well as the secular–religious spectrum, reveals continuous disparities in prenatal testing uptake and decision-making about pregnancy termination [191,192,193]. Secular groups are more likely to accept screenings and diagnostic alternatives, whereas religious societies are more cautious and prioritise value-sensitive, non-directive counselling, confidentiality, and provider trust. Israeli studies also show how societal norms, perceptions of impairment, and collective family decision-making influence responses to preconception and prenatal genetics, particularly among women who conceived through IVF [194,195,196,197]. These findings are important in Eastern European settings because they demonstrate that acceptability is determined not only by access or affordability but also by how programmes match religious views, societal values, and local leadership [193,198].

In Eastern Europe, particularly Bulgaria, cultural and religious traditions, health literacy, and community trust all influence acceptability of preconception care and genetic screening. Programmes are more effective when they (a) emphasise voluntariness, informed consent, and confidentiality; [199,200,201] (b) provide culturally competent, person-centred counselling (allowing for longer consultations, interpreters, and in-language materials); [202,203] and (c) involve trusted community and religious leaders as partners rather than mere stakeholders [204]. Prioritising underprivileged groups and explicitly involving partners and males in counselling and decision-making might improve informed choice, minimise stigma, and promote equitable uptake [37,205,206].

Potential human rights hazards related to PCC include the loss of reproductive liberty, privacy violations, and discrimination—particularly if promotional activities generate social pressure to comply with “normative” reproductive choices or undertake genetic screening [127,204,205,206,207]. PCC can also medicalise the pre-pregnancy period and encourage gender stereotyping by assuming that everyone has the desire or capacity for pregnancy [37,208]. Nordic welfare traditions, while strongly orientated towards universalism and equity, may be wary of subtle forms of social pressure or surveillance justified in the name of population health; meanwhile, north-west European countries (e.g., the Netherlands, England, France, and Germany) demonstrate more varied balances between reproductive autonomy, human dignity, and disability rights, as reflected in different regulatory approaches and ethical debates [127,199,207]. For Bulgaria, the following safeguards should be explicit and operationalised: strict voluntariness; clear and comprehensible information; strong confidentiality; non-directive and non-stigmatising communication; routine ethics oversight; and ongoing monitoring for unintended harms (for example, gender bias or discrimination). All proposed pathways are anchored in GDPR-consistent data protection, explicit informed consent, and non-discrimination safeguards.

## 8. Future Directions

The successful incorporation of PCC into Bulgaria’s health system necessitates a coordinated multi-level strategy that integrates system reforms, provider training, public engagement, and equitable access to genetic services. As a single-payer system, NHIF financing and GP-centred primary care determine access and referral pathways. Limited reimbursement for preconception services and concentration of genetics capacity in academic/private centres constrain equitable uptake. Enhancing provider capacity is a primary objective: formulating national clinical guidelines in accordance with WHO and FIGO recommendations and executing continuing medical education for GPs and OB/GYNs will facilitate standardised and consistent delivery of PCC.

The promotion of public awareness and community engagement is essential to normalise PCC as a standard aspect of reproductive health. Outreach efforts must prioritise socioeconomically disadvantaged and rural populations, including Roma communities, where health literacy and access are significantly constrained. ECS is a strategic long-term objective that should be implemented gradually, starting with voluntary testing for high-risk populations, alongside partial public co-funding and culturally appropriate genetic counselling. This phased approach integrates clinical utility with ethical safeguards and considerations of equity.

The integration of digital health innovations, such as teleconsultation services, mobile applications, and clinical decision-support tools, should be piloted and assessed for their potential to improve behavioural engagement and broaden PCC coverage. Integrating these digital platforms into the current health system will enhance proactive risk assessment, patient follow-up, and national data collection, thereby contributing to the establishment of a sustainable, evidence-based PCC framework in Bulgaria (Table 3).

## 9. Limitations

This review was confined to English-language sources, potentially omitting relevant national-level information from Bulgaria and other non-English-speaking nations. Moreover, a significant portion of the Bulgarian evidence originates from small-scale or qualitative investigations, thereby constraining its national representativeness. Moreover, using high-quality grey literature may lead to inconsistencies in methodological rigour among these sources, which can adversely affect the dependability of the conclusions. The existing literature’s inadequate address of systemic, cultural, and financial disparities may limit the applicability of international implementation strategies for Bulgaria. Economic and epidemiological [7,8,24,35,36,37,38,39,40,41,42,43,44,45,46,47,48,49,50,51,52,53,54,55,56,57,58,59,60,61,62,63,64,65,66,67,68,69,70,71,72,73,74,75,76,77,78,79,80,81,82,83,84,85,86,87,88,89,90,91,92,93,94,95,96,97,98,99,100,101,102,103,104,105,106,107,108,109,110,111,112,113,114,115,116,117,118,119,120,121,122,123,124,125,126,127,128,129,130,131,132,133,134,135,136,137,138,139,140,141,142,143,144,145,146,147,148] modelling to forecast ECS/PCC impact was beyond our narrative scope and should be prioritised in future, using Bulgarian prevalence and cost data. Future research should prioritise nationally representative studies, incorporate local-language resources, and conduct a thorough evaluation of pilot PCC programmes.

## 10. Conclusions

PCC serves as a fundamental component of preventive reproductive health, connecting early medical, behavioural, and genetic interventions to enhanced maternal and neonatal outcomes. International evidence indicates that organised, digitally facilitated PCC strategies diminish avoidable complications and improve health equity.

In Bulgaria, progress is impeded by fragmented service delivery, the absence of a national strategy, limited provider capacity, and minimal public engagement. Integrating PCC into routine primary care requires the establishment of national guidelines, professional training, digital innovation, and a systematic approach to genetic screening. The proposed changes aim to enhance maternal and child health, promote reproductive autonomy, bolster demographic resilience, and ensure the long-term sustainability of the Bulgarian healthcare system. PCC integration into routine primary care, combined with culturally competent genetic counselling and progressive reimbursement mechanisms, has the potential to enhance reproductive equity and demographic resilience in Bulgaria.

## Figures and Tables

**Table 1:** PCC Strategies in European Countries.

Country	[14,38,39,40,41,42,43,44,45,46,47,48,49,50,51,52,53,54,55,56,57,58,59,60,61,62,63,64,65,66,67,68,69,70,71,72,73,74,75,76,77,78,79,80,81,82,83,84,85,86,87,88,89,90,91,92,93,94,95,96,97,98,99,100,101,102,103,104,105,106,107,108,109,110,111,112,113,114,115,116,117,118,119,120,121,122,123,124,125,126,127,128,129,130,131,132,133,134,135,136,137,138,139,140,141,142,143,144,145,146,147,148,149,150,151,152,153,154] PCC Policy	Guidelines/Recommendations	Services & Programmes
**Austria** [38,39,40,41,42]	Integrated into maternal and child health policies; supported through the “Eltern-Kind-Pass” scheme covering pre-pregnancy and antenatal care.	National recommendations on folic acid supplementation, healthy lifestyle, and vaccination before conception.	“Eltern-Kind-Pass” with PCC consultation; public health centres, hospitals, and family planning associations provide counselling.
**Belgium (Flanders)** [43,44,45,46,47,48]	Regional PCC policy focuses on reproductive health, folic acid promotion, and counselling.	Regional guidelines on nutrition, lifestyle, and vaccination; included in maternal care protocols.	PCC consultations through general practitioners, midwives, and specialized clinics; public awareness via BelPreg and national campaigns.
**Bulgaria** [14,49,50,51]	PCC elements integrated into reproductive health and maternal health strategies; emphasis on vulnerable groups through EU Child Guarantee initiatives.	Recommendations for folic acid, prevention of genetic disorders, vaccination, and lifestyle modification before conception.	Provided in maternal and child health consultations; NGOs and women’s health associations support awareness campaigns.
**Croatia** [52,53,54]	PCC integrated into maternal and reproductive health strategies, with a focus on prenatal screening and folic acid supplementation.	Guidelines on folic acid, vaccination, and prevention of congenital anomalies; limited PCC-specific national strategy.	Public health institutes, maternity hospitals, and NGOs provide information and counselling services.
**Cyprus** [55,56,57,58,59]	PCC addressed in maternal and child health policies; includes nutrition, vaccination, and chronic disease management before conception.	National recommendations on folic acid and rubella vaccination; targeted programs in maternal and child welfare centres.	Provided through Maternal and Child Welfare Centres and hospitals; outreach programs in rural areas; includes services in Northern Cyprus.
**Czech Republic** [60,61,62,63]	PCC addressed through maternal health programs and folic acid fortification policies; integrated into reproductive health monitoring.	Guidelines on folic acid supplementation and lifestyle modification before conception.	Services offered via general practitioners, obstetrician–gynaecologists (OB/GYNs), and maternity hospitals; supported by awareness campaigns.
**Denmark** [64,65,66,67,68,69]	PCC is part of primary healthcare, with strong integration into reproductive health counselling.	National recommendations on folic acid, vaccination, and chronic disease management before conception.	PCC consultations offered in general practice, hospital outpatient clinics, and municipal health centres; midwives and public health nurses are key providers.
**Estonia** [70,71,72,73,74,75]	PCC addressed through maternal health policy and digital health integration.	Recommendations on folic acid, vaccination, and lifestyle; electronic health record facilitates PCC delivery.	PCC consultations at women’s clinics and primary care; integration with digital services (E-Health Records, TEHIK).
**Finland** [76,77,78,79,80,81,82]	PCC embedded in maternity and child health clinic (Neuvola) system.	National guidelines on folic acid, vaccinations, healthy weight, and chronic disease control before conception.	Neuvola clinics provide universal PCC services, including counselling, screening, and follow-up; midwives play a central role.
**France** [83,84,85,86]	PCC integrated into reproductive health policies targeted at risk groups and couples planning pregnancy.	Guidelines on folic acid, vaccinations, and lifestyle; recommendations for pre-pregnancy medical check-ups.	PCC consultations available in general practice, maternity units, and family planning centres.
**Germany** [87,88,89,90,91,92]	PCC included in maternal health framework and preventive healthcare legislation.	Recommendations on folic acid, rubella immunity, chronic disease management, and genetic counselling.	Provided by OB/GYNs, family doctors, and midwives; integrated with digital health applications and preventive programs.
**Greece** [93,94,95]	PCC integrated into reproductive health policy; addressed mainly through gynaecological and maternal health services.	Guidelines on folic acid, vaccinations, nutrition, and chronic disease control.	Services through OB/GYNs, maternity hospitals, and public health units; awareness campaigns for reproductive-age women.
**Hungary** [63,96,97,98]	PCC integrated into reproductive health services with long-standing PCC programs.	Guidelines include folic acid, genetic counselling, vaccination, and chronic disease control.	Provided in specialized PCC clinics, family practices, and public health services.
**Ireland** [99,100,101,102]	PCC addressed through national maternity strategy; integrated in primary care and hospital services.	Guidelines include folic acid, vaccinations, lifestyle counselling, and chronic disease management.	Provided through GPs, midwives, and hospital outpatient clinics; supported by public awareness initiatives.
**Italy** [44,103,104,105,106]	PCC integrated into maternal and reproductive health policy.	Guidelines on folic acid, lifestyle, vaccination, and genetic counselling.	Provided through public health centres, gynaecology clinics, and family doctors; national campaigns for folic acid awareness.
**Latvia** [107,108,109,110,111]	PCC included in reproductive and maternal health policies.	Guidelines on folic acid, vaccination, and nutrition for women before conception.	Provided through primary care, gynaecologists, and public health centres; targeted programs for at-risk populations.
**Lithuania** [112,113,114,115,116]	PCC is integrated into national health programme and reproductive health policy.	Recommendations on folic acid, healthy lifestyle, and screening for chronic diseases.	Services in primary care and maternal health centres; NGO support for awareness campaigns.
**Luxembourg** [117,118,119]	PCC integrated into reproductive health strategies with universal access to contraception and preventive services.	Recommendations on folic acid, vaccination, and healthy lifestyle before conception.	Services provided by GPs, OB/GYNs, and family planning centres; contraceptives reimbursed 100%.
**Malta** [120,121,122,123,124]	PCC integrated into sexual and reproductive health strategies.	Guidelines include folic acid supplementation, vaccination, and chronic disease management.	Provided through public health centres, maternal clinics, and outreach services; midwives offer PCC counselling.
**Netherlands** [44,125,126,127,128,129,130,131]	PCC integrated into primary and reproductive healthcare policy.	Guidelines on folic acid, vaccination, lifestyle, and chronic disease management.	Provided through GPs, midwives, and online tools like ZwangerWijzer; local and national PCC initiatives.
**Poland** [63,132,133]	PCC is part of national reproductive health strategy.	Recommendations on folic acid, vaccination, and chronic disease control.	Provided through OB/GYNs, primary care, and public health programs; folic acid supplementation campaigns ongoing.
**Portugal** [134,135,136]	PCC is integrated into national maternal health programmes.	Guidelines on folic acid, vaccination, and lifestyle before conception; maximum deadlines for PCC consultations established.	PCC is provided in primary care, maternity hospitals, and public health services.
**Romania** [137,138,139]	PCC included in reproductive health policy; targeted programs for maternal mortality reduction.	Recommendations on folic acid, vaccination, and chronic disease management before conception.	Provided through primary care, OB/GYNs, and maternal health clinics; NGO involvement in awareness campaigns.
**Slovakia** [63,140,141,142]	PCC is integrated into maternal and child health strategies.	Guidelines on folic acid, vaccination, and lifestyle modification before conception.	Services in primary care, OB/GYNs, and maternity hospitals; supported by public health initiatives.
**Slovenia** [143,144,145,146]	PCC is integrated into reproductive and maternal health strategies.	Recommendations on folic acid, vaccination, and healthy lifestyle; emphasis on health literacy.	Provided in primary care, maternity hospitals, and public health units.
**Spain** [147,150]	PCC integrated into maternal health policy.	Guidelines on folic acid, healthy diet, vaccination, and prevention of congenital disorders.	Services through primary care, gynaecology clinics, and public health campaigns; clinical guidelines accessible nationally.
**Sweden** [44,151,152,153,154,155]	PCC addressed maternal and reproductive health policy with focus on health equity.	Guidelines on folic acid, vaccination, genetic carrier screening, and chronic disease management.	Provided in primary care, maternity units, and public health clinics; research-based interventions inform practice.

**Table 2:** Critical Analysis of International PCC Models and Applicability to Bulgaria.

Model	Strengths	Limitations	Applicability in Bulgaria
**Dutch model—PCC integrated into primary care with digital tools**	High population coverage; strong eHealth integration	Requires well-developed primary care network and robust IT infrastructure	Partially applicable; would require GP training, patient engagement strategies, and investment in digital systems
**UK/NICE—formalised guidelines and broad interventions**	Standardised protocols; comprehensive coverage of >30 risk factors; strong guideline authority	Limited real-world implementation (~7.6% documented PCC uptake)	Adaptable in terms of guidelines and provider training; would require incentives for uptake
**LMIC community-based—mobile clinics and peer education**	Cost-effective; reaches underserved and vulnerable populations	Dependent on continuous funding; limited sustainability without public sector support	Highly applicable for Roma communities and rural/remote areas
**Middle East—mandatory premarital screening**	Demonstrated reduction in prevalence of certain autosomal recessive conditions	Ethical and human rights concerns; risk of stigma and discrimination	Mandatory model not recommended; voluntary, culturally sensitive targeted screening may be suitable

Source: Adapted from Maas et al., 2022 [181]; Gregg et al., 2021 [159]; WHO [7,8].

**Table 3:** Proposed Policy Recommendations for Implementing PCC in Bulgaria.

Timeframe	Policy Recommendations
**Short-term (1–2 years)**	Develop national PCC guidelines; implement targeted training for GPs and OB/GYNs; launch pilot digital PCC platforms; initiate voluntary, targeted genetic screening for high-risk populations
**Long-term (5+ years)**	Secure PCC coverage under the National Health Insurance Fund (NHIF); establish a national PCC registry and continuous data monitoring system; introduce expanded carrier screening (ECS) with partial public funding; integrate PCC education into school curricula and community health programmes

Source: Adapted from [7,8,24,35,36,37,38,39,40,41,42,43,44,45,46,47,48,49,50,51,52,53,54,55,56,57,58,59,60,61,62,63,64,65,66,67,68,69,70,71,72,73,74,75,76,77,78,79,80,81,82,83,84,85,86,87,88,89,90,91,92,93,94,95,96,97,98,99,100,101,102,103,104,105,106,107,108,109,110,111,112,113,114,115,116,117,118,119,120,121,122,123,124,125,126,127,128,129,130,131,132,133,134,135,136,137,138,139,140,141,142,143,144,145,146,147,148].

## Data Availability

The data presented in this study are available on request from the corresponding author.

## Abbreviations

The following abbreviations are used in this manuscript:

PCC	Preconception care
ECS	Expanded carrier screening
WHO	World Health Organisation
GPs	General practitioners
OB/GYNs	Obstetrician–gynaecologists
NGO	Non-Governmental Organization
LMICs	low- and middle-income countries
ACOG	American College of Obstetricians and Gynaecologists
FIGO	International Federation of Gynaecology and Obstetrics
BFPA	Bulgarian Family Planning and Sexual Health Association
